# Spontaneous ilio-psoas haematomas (IPHs): a warning for COVID-19 inpatients

**DOI:** 10.1080/07853890.2021.1875498

**Published:** 2021-01-25

**Authors:** Alessandra Vergori, Elisa Pianura, Patrizia Lorenzini, Alessandra D’Abramo, Federica Di Stefano, Susanna Grisetti, Serena Vita, Carmela Pinnetti, Davide Roberto Donno, Maria Cristina Marini, Emanuele Nicastri, Stefania Ianniello, Andrea Antinori, on behalf of the ReCOVeRI Study Group

**Affiliations:** a HIV/AIDS Unit, National Institute for Infectious Diseases Lazzaro Spallanzani IRCCS, Rome, Italy; b Radiology Unit, National Institute for Infectious Diseases Lazzaro Spallanzani IRCCS, Rome, Italy; c Emerging Infectious Diseases Unit, National Institute for Infectious Diseases Lazzaro Spallanzani IRCCS, Rome, Italy; d Severe and Immune-depression Associated Infectious Diseases Unit, National Institute for Infectious Diseases Lazzaro Spallanzani IRCCS, Rome, Italy; e Intensive Care Unit, National Institute for Infectious Diseases Lazzaro Spallanzani IRCCS, Rome, Italy

**Keywords:** Ilio-psoas haematoma, heparin, COVID-19

## Abstract

**Introduction:**

Critically ill patients with COVID-19 are at increased risk of developing a hypercoagulable state due to haemostatic changes directly related to the SARS-CoV-2 infection or to the consequence of the cytokine storm. Anticoagulation is now recommended to reduce the thrombotic risk. Ilio-psoas haematoma (IPH) is a potentially lethal condition that can arise during the hospitalization, especially in intensive care units (ICUs) and frequently reported as a complication of anticoagulation treatment.

**Materials and methods:**

We report a case series of seven subjects with SARS-CoV-2 pneumonia complicated by Ilio-psoas haematomas (IPHs) at our COVID-Hospital in Rome, Italy.

**Results:**

Over the observation period, 925 subjects with confirmed SARS-CoV-2 infection were admitted to our COVID-hospital. Among them, we found seven spontaneous IPHs with an incidence of 7.6 cases per 1000 hospitalization. All the reported cases had a severe manifestation of COVID-19 pneumonia, with at least one comorbidity and 5/7 were on treatment with low weight molecular heparin for micro or macro pulmonary thrombosis.

**Conclusions:**

Given the indications to prescribe anticoagulant therapy in COVID-19 and the lack of solid evidences on the optimal dose and duration, it is important to be aware of the iliopsoas haematoma as a potentially serious complication in COVID-19 inpatients.

**KEY MESSAGE**
- Critically ill patients with COVID-19 are at increased risk of hypercoagulability state and anticoagulation therapy is recommended.
- Ilio-psoas haematoma (IPH) is found to be a complication of anticoagulation regimen especially in severe COVID-19 cases.
- An incidence of 7.6 cases per 1000 admission of IPHs was reported.
- Hypoesthesia of the lower limbs, pain triggered by femoral rotation, hypovolaemia and anaemia are the most common symptoms and signs of IPHs that should alert physician.

## Introduction

Critically ill patients with coronavirus disease-2019 (COVID-19) are at increased risk of developing a hypercoagulable state [1].

The pathophysiology behind this phenomenon has been suspected to be a result of haemostatic changes that might be direct effect of severe acute respiratory syndrome-coronavirus-2 (SARS-CoV-2) or a consequence of a cytokine storm that alters the onset of the systemic inflammatory response syndrome (SIRS), as observed in other viral disease [2]. To hinder this hypercoagulable state, COVID-19 patients should be properly anticoagulated to reduce the thrombotic risk [3–8].

IPH is a potentially lethal condition frequently reported as a complication of anticoagulation therapy that can arise during the hospitalization, especially in intensive care units (ICUs) [9]. To date, a single case of spontaneous IPH in a COVID-19 patient has been reported [10]. Here, we report seven cases of spontaneous IPHs occurring in patients with severe COVID-19 pneumonia admitted to our Institute.

## Materials and methods

### Study population and setting

We consecutively included all subjects with a microbiologically confirmed SARS-CoV-2 infection who were admitted to the National Institute for Infectious Diseases IRCCS Lazzaro Spallanzani in Rome, Italy, between 1 March 2020 and 30 October 2020. Medical history, demographic and clinical data were collected through review of medical records. Data have been collected for the ReCOVeRI Study, a registry on COVID-19 for clinical Research of the National Institute for Infectious Diseases L. Spallanzani, approved by the Ethical Committee of the National Institute for Infectious Diseases L. Spallanzani IRCCS (number 164, 26 June 2020). Laboratory and radiologic assessments during the hospital stay were performed by the treating physician according to the hospital operative procedure. All patients gave informed consent for collecting personal data for research purposes.

### Definitions

A confirmed case of COVID-19 was defined by a positive real-time reverse-transcription PCR (RT-PCR) assay for SARS-CoV-2 on nasopharyngeal swab and/or a positive serology for SARS-CoV-2 (positive immunoglobulin (Ig) G or M or A for SARS-CoV-2).

Severe disease was defined as clinical signs of pneumonia plus one of the following: respiratory rate greater than 30 breaths per min, severe respiratory distress or oxygen saturation less than 90% on room air [11].

Hyperinflammation syndrome was defined as having at least two among D-dimer above 1000 ng/mL, ferritin above 500 mcg/L, LDH above 300 UI/L and lymphocyte count below 1000 cell/mm^3^ [1]. Ilio-psoas haematoma was diagnosed by using computerized tomography (CT)-scan or magnetic resonance imaging (MRI).

All CT scans were performed on a multi-detector row CT scanner (Bright Speed, General Electric Medical Systems, Milwaukee, WI) using 120 kV pp, 250 mA, pitch of 1.375, gantry rotation time of 0.6 s. A chest and abdomen CT was performed from the apex of lung to symphysis pubis before and after injection of iodinated contrast media into a peripheral vein with three-phase arterial, venous and delayed phase. The baseline scan of the thorax was reconstructed with slice thicknesses of 0.625 mm and spacing of 1 mm with high contrast resolution algorithm. The contrast media scan of the thorax and abdomen was reconstructed with slice thicknesses of 1.25 mm and spacing of 1 mm, completed with multiplanar reconstructions (MPR and Mip). The MRI scan was performed on HDx scanner (General Electric Medical Systems, Milwaukee, WI), 1.5 T, with T1 and T2 weighted sequences also after spectral fat subtraction and paramagnetic contrast media.

## Results

Over the observation period, 925 subjects with confirmed SARS-CoV-2 infection were admitted to our COVID-hospital. Among them, we observed seven spontaneous IPHs with an incidence of 7.6 cases per 1000 hospitalization.

### Clinical characteristics and outcome

Main demographic and clinical characteristics and outcomes are summarized in Table 1.

**Table 1.** Characteristics of the reported cases.

	Patient 1	Patient 2	Patient 3	Patient 4	Patient 5	Patient 6	Patient 7
Age	80	75	94	55	64	72	66
Gender	M	F	F	M	F	M	F
Comorbidities	Atrial fibrillation; COPD	Hypertension; asthma	Diabetes, ischaemic heart disease, hypertension, cognitive impairment, bedridden syndrome	Not reported	Ischaemic heart disease, obesity	Diabetes, hypertension	Thyroid disease
Clinical presentation at admission							
Days from symptoms onset to admission	14	5	2	14	7	7	7
Symptoms	Arthralgia, asthenia, dyspnoea, fever, cough	Headache, dyspnoea, fever, cough, myalgia	Fever, cough	Fever, cough	Fever, cough	Fever, cough, dyspnoea	Fever, dysgeusia, cough
SpO2 at admission on room air	90%	96%	95%	93%	91%	90%	92%
Days from admission to IPH	9	25	14	5	38	30	18
Days from admission to prophylactic anticoagulation start	1	9	0	0	0	0	0
Length of hospital stay (days)	20	50	36	34	–	70	29
Exitus	Died	Discharged	Discharged	Discharged	Still admitted	Discharged	Discharged
Inflammatory Index at admission							
Haemoglobin, g/dL	13.9	11.6	12.8	7.4	11	12.3	13.8
Platelets, ×10^3^/mm^3^	121	142	185	176	255	193	350
Lymphocytes, cell ×10^3^/mm^3^	4.2	9.6	12.2	15.0	8.1	7.5	12.21
LDH, UI/L	404	576	254	249	325	205	433
D-dimer, ng/mL	796	740	551	1373	608	10,850	901
Fibrinogen, mg/dL	798	487	47	314	824	531	706
ferritin, ng/mL	2167	4754	1113	548	303	369	534
PT, INR	0.93	1.08	1.23	1.18	1.06	1.12	1.21
PTT, sec	48.4	35.7	48.7	33.1	24.6	26.3	26.6
C-reactive protein, md/dL	15.78	8.62	33.1	16.2	12.9	4.5	6.6
Inflammatory index at ilio-psoas haematoma							
Haemoglobin, g/dL	8.7	6.8	10.5	8	6.9	4.9	8.8
Platelet, ×10^3^/mm^3^	234	91	295	173	492	217	410
Lymphocytes, cell ×10^3^/mm^3^	6.3	10.3	13.8	9.4	16.1	13.4	11.6
LDH, UI/L	377	441	254	208	410	–	417
D-dimer, ng/mL	796	2033	1287	1517	728	8000	NA
Fibrinogen, mg/dL	351	241	685	361	382	450	
Ferritin, ng/mL	2167	3506	903	1010	1000	NA	NA
PT, INR	1.06	1.13	0.94	1.33	1.13	1.18	
aPTT, s	29.3	16.6	38.6	33.9	40.7	28.2	
C-reactive protein, md/dL	0.62	1.52	1.97	12.23	2.28	13	4.73
Treatment during hospitalization							
HCQ	No	Yes	No	No	No	No	No
Lopinavir/ritonavir	Yes	Yes	Yes	No	No	No	No
Remdesivir	–	–	Yes	–	Yes	Yes	Yes
Steroids	Methyl-prednisolone	Methyl-prednisolone	No	No	No	Desametasone	Desametasone
LMWH prophylaxis	No	Yes	Yes	Yes	Yes	Yes	Yes
LMWH treatment	8000 UI ×2/day	No	No	6000 UI ×2/day	6000 UI ×2/day	6000 UI ×2/day	6000 UI ×2/day
Need for oxygen supplement	Yes (cPAP)	Yes (cPAP)	Yes (VM with FiO2 40%)	Yes (OTI)	Yes (OTI)	Yes (NIV)	Yes (OTI)

IPH: ilio-psoas haematoma; LDH: lactic dehydrogenase; NA: not available; PT: prothrombin time; INR: international normalized ratio; aPTT: activated partial thromboplastin time; HCQ: hydroxychloroquine; LMWH: low molecular weight heparin; cPAP: continuous positive airway pressure; VM: venturi mask; OTI: orotracheal intubation; NIV: non-invasive ventilation.

Four patients were female with an age ranging between 65 and 80 years and a median body mass index of 28 (IQR 25–32). At the time of COVID-19 diagnosis, all patients were diagnosed with pneumonia and had at least one comorbidity; among them, three patients had hypertension, two patients had diabetes and one patient a chronic obstructive pulmonary disease (COPD). The median hospital stay was 34 days (IQR 30–36). Severe clinical presentation was observed in four patients with an admission oxygen saturation (SpO2) 93% on room air (IQR 90–98). The arterial oxygen partial pressure (PaO2 in mmHg) to fractional inspired oxygen (PaO2/FiO2) ratio was between 200 mmHg and 300 mmHg and it required supplemental oxygen therapy with non-invasive ventilation by using continuous positive airway pressure (cPAP). Three patients required invasive mechanical ventilation with orotracheal intubation (OTI) and admission to the ICU. Chest CT scan performed at admission, showed bilateral ground-glass opacities (GGOs) and sub-segmental consolidations, mostly located in the peripheral zone. Moreover, other imaging features such as linear opacities, “crazy-paving” pattern, the “reverse halo sign” and subsegmental vessel enlargement were described. In particular, the vessel enlargement was described close to the GGOs, which is compatible with thrombo-inflammatory processes. The contrast enhancement scan showed in five out of seven thrombosis-mediated micro-perfusion defects in peripheral pulmonary vessels.

All patients received prophylactic low weight molecular heparin (LWMH) at admission, with the exception of one patient who started anticoagulant therapy with LWMH for pulmonary embolism previously diagnosed at the emergency department. The heparin dosages were modified according to clinical worsening and to the diagnosis of micro or macro pulmonary thromboembolic events. Only one patient was taking antiaggregants medications discontinued as soon as the diagnosis of IPH was made.

Overall, steroid therapy was administered in five patients. Specifically, concerning SARS-CoV-2 therapy, four patients received oral lopinavir/ritonavir (LPV/r, 400/100 mg twice per day for 14 days), one of them received also oral hydroxychloroquine (200 mg twice per day for 10 days); four patients were treated with intravenous remdesivir (200 mg on day one followed by 100 mg since day 2 to day 10), combined with intravenous dexamethasone (6 mg once daily for 10 days) in three of them. Inflammation and coagulation parameters are shown in Table 1. Briefly, all patients had an hyperinflammation pattern with a median ferritin level of 548 pg/mL (IQR 369–2167 pg/mL) and C-reactive protein (CRP) of 8.6 mg/dL (IQR 6.6–15.8 mg/dL) and a D-dimer of 796 ng/mL (IQR 608–1373 ng/mL).

During the hospitalization, after a median of 35 (IQR 29–50) days, signs of neurologic compression with hypoesthesia of the lower limbs and general signs of hypovolaemia and anaemia were always present. A CT scan of the abdomen was performed revealing iliopsoas haematoma in all patients apart from one subject studied by performing an MRI.

The patients were treated in a conservative manner, resuscitated, according to clinical judgement, with intravenous fluid, transfusions of red blood cells and other supportive measures; only one patient, haemodynamically unstable, underwent arterial embolization.

Five patients were discharged with no long-lasting complications, one patient is still hospitalized and one patient died.

## Imaging

The differential diagnosis of pelvic mass of the abdominal wall includes the most frequent pathologies as sarcoma, haematoma and abscess. Mass signal characteristics, vascular pattern during and after intravenous injection of contrast media and the presence of other elements (e.g. small intralesional calcifications) might help the radiologist to perform a correct diagnosis.

In our cases, a non-enhanced CT scan of the abdomen showed diffuse enlargement as well as heterogeneous density of the ilio-psoas muscle with an area of high density or fluid-fluid level for subacute haematoma. The absence of a contiguous mass with a vascular pattern led to rule out a diagnosis of haemorrhagic sarcoma.

The size of the haematomas found ranged from a minimum of 4–10 cm. In four patients, the dimensions exceeded 12 cm of transverse diameter and longitudinal extension of about 10 cm.

**Figure 1.**
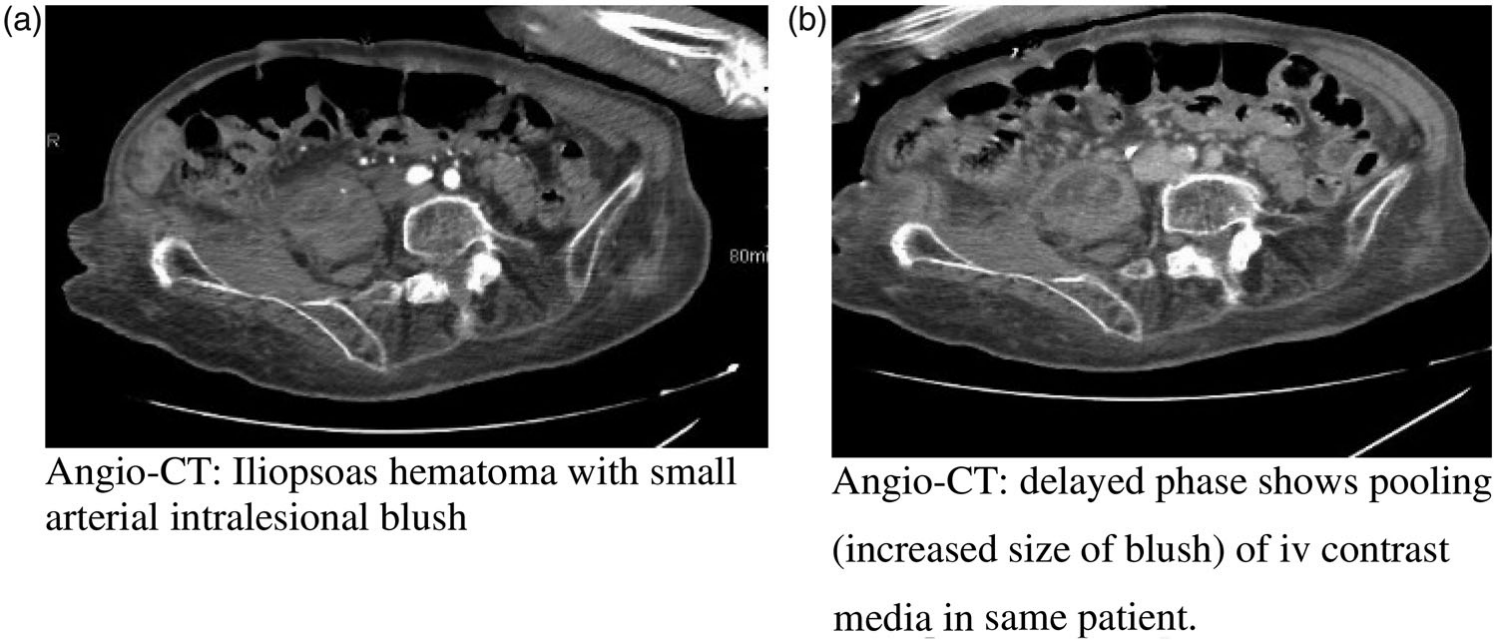
(a) Arterial phase CT of right haematoma and (b) delayed phase CT of right haematoma.

In two cases, the CT angiogram showed multiple streaks of contrast blush; delayed phase shows pooling (increased size of blush) of intravenous contrast media (Figure 1(a,b)). Contrast media blush, lack of internal fluid density and marginal enhancing component may exclude the diagnosis of iliopsoas abscess and raises the possibility of subacute haematoma.

**Figure 2.**
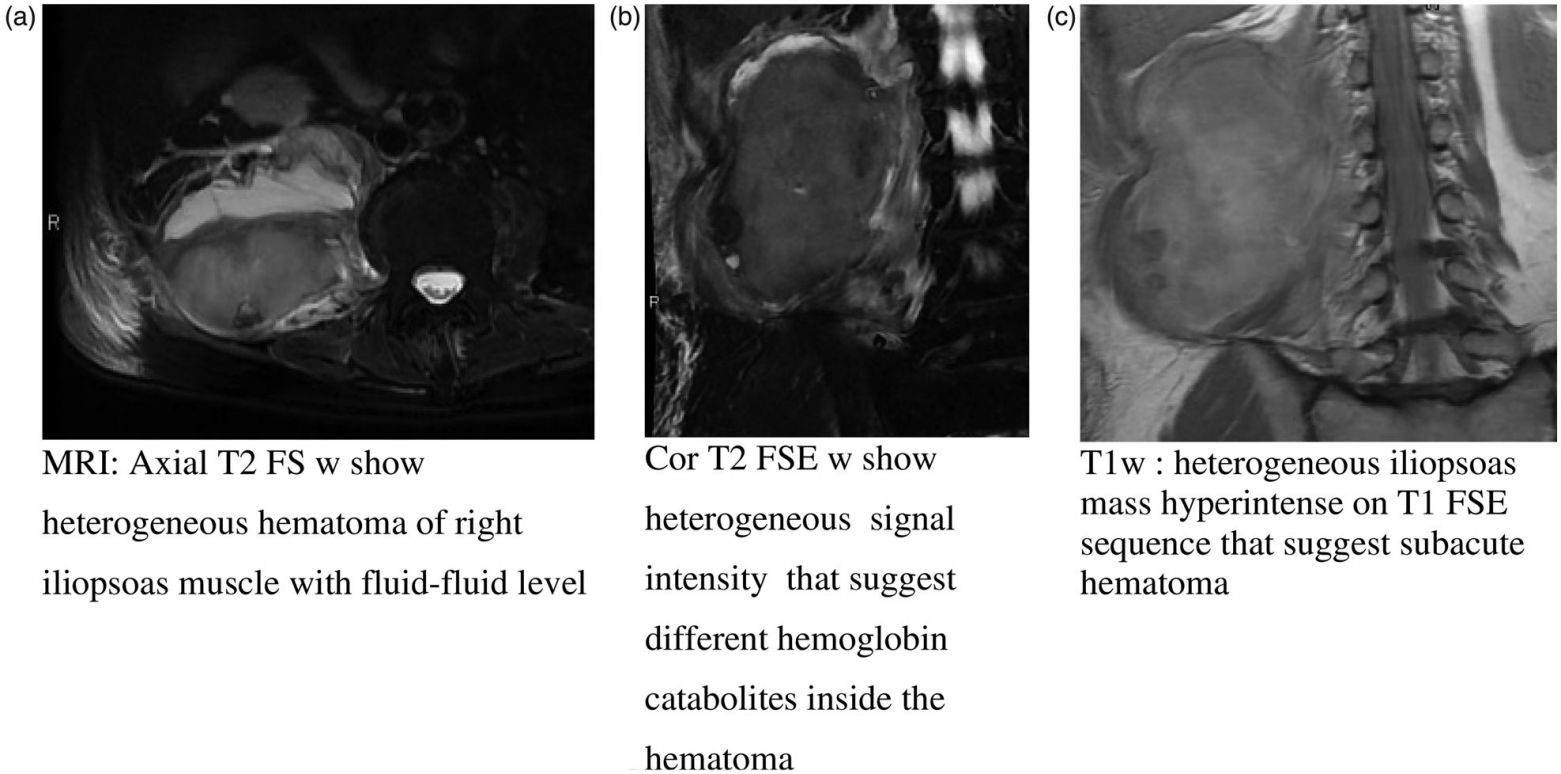
(a) MRI of right iliopsoas haematoma, (b) MRI of right iliopsoas haematoma and (c) MRI of right iliopsoas haematoma.

In one case, MRI study was performed and showed a heterogeneous iliopsoas mass, with normal diffusion-weighted imaging (DWI) pattern and hyperintense areas on T1- and T2-weighted images for haemoglobin catabolites (metaHb) for subacute haematoma (Figure 2(a–c)).

The MRI findings correlated with the evolution of the haematoma:

- *Acute haematoma*: Iso-hypointense in T1-weighted (w) or slightly hypointense to muscle and hypo-hyperintense in T2 weighted (w).
- *Subacute haematoma*: Hyperintense in T1w and T2w; high intensity rim, higher intensity peripheral zone and lower intensity core in T1w and relatively higher signal from core to periphery in T2w.
- *Chronic haematoma*: Hypointense rim in T1w and T2w.

## Discussion

Spontaneous IPH is defined as a retroperitoneal collection of blood involving the ilio-psoas muscle. Few studies evaluated the incidence of spontaneous IPHs in patients undergoing anticoagulation therapy that has been reported ranging from 0.1 to 0.6% [12]. Recently, a retrospective study in no COVID patients showed an incidence of IPHs of 3.8 cases per 1000 admission in ICUs [9] and our incidence of 7.6 cases over 1000 hospitalization, is higher than that previously reported. Risk factors in our small case series are the same reported in literature, as age, anticoagulation, a high body mass index, comorbidities such as hypertension and diabetes [9,13,14], moreover, our patients had an activated partial thromboplastin time (aPTT) above the therapeutic range at the moment of the diagnosis of the IPHs. Disseminated intravascular coagulopathy (DIC) and increased aPTT are both independent predictors of unfavourable prognosis [9]. The precise pathogenesis of retroperitoneal bleeding is unknown, it is most commonly reported as a complication of anticoagulation and, more rarely, in the setting of a clotting disorder or traumatic injury during the patients’ mobilization in the prone position [15–19].

Due to the anatomical proximity of these muscles to the lumbar plexus, it is hypothesized that retroperitoneal pre-existing microvascular atherosclerosis could increase sensitivity to rupture and microtrauma such as cough or vomiting could also lead to retroperitoneal bleeding [20].

It is well known that one of the causes of mortality in COVID-19 patients is venous thromboembolism (VTE) as evidenced by altered coagulation profile like elevated D-dimers [1,20].

This hypercoagulable phenomenon is due to the increased pro-inflammatory cytokines leading to atherosclerotic changes through local inflammation, microvascular thrombi and haemodynamic changes with multiorgan failure and death [1]. For these reasons, the administration of prophylactic or therapeutic anticoagulant agents is recommended [3–8]. Five cases among those we reported were fully treated with low molecular weight heparin for pulmonary micro-thrombosis.

Anticoagulation treatment and additional anti-platelet medications, increase the risk of major bleeding complications like retroperitoneal haemorrhage [9,10]. In a French ICU, 19 out of 92 (21%) COVID-19 patients on full anticoagulant treatment had 22 haemorrhagic events, and five of them were gastrointestinal [21].

Regarding IPHs, the optimal treatment remains controversial; however, initial treatment consists of discontinuation of anticoagulant agents, transfusion therapy, volume resuscitation and supportive measures; haemodynamically unstable patients, according to expert clinical judgement, can be treated with arterial embolization as it is minimally invasive with quick therapeutic effect when compared with surgical treatment.

Furthermore, stopping anticoagulation in case of IPHs and of a documented pulmonary micro-thrombosis in COVID-19 could be life-threatening.

Given the indications to prescribe anticoagulation in COVID-19 and the lack of solid evidences on the optimal dose and duration, specifically in micro-thrombosis, it is important to be aware of the iliopsoas haematoma as a potentially serious complication. As there is no consensus on therapeutical management of IPHs, each decision (i.e. conservative treatment, embolization, surgical or CT scan-guided haematoma’s drainage) should be made according to the clinical stability of the patients and by weighting risks and benefits.

We definitely need more studies in order to establish which is the optimal heparin dose and how long it should be continued in micro-thrombosis, as anticoagulation may increase the risk of major and potentially fatal bleeding.

## Data Availability

Data available on request from the authors.

## Appendix

The authors gratefully acknowledge nurse staff, all the patients and all members of the ReCOVeRI Study Group: Maria Alessandra Abbonizio, Amina Abdeddaim, Elisabetta Agostini, Chiara Agrati, Fabrizio Albarello, Gioia Amadei, Alessandra Amendola, Andrea Antinori, Maria Assunta Antonica, Mario Antonini, Tommaso Ascoli Bartoli, Francesco Baldini, Raffaella Barbaro, Barbara Bartolini, Rita Bellagamba, Martina Benigni, Nazario Bevilacqua, Gianluigi Biava, Michele Bibas, Licia Bordi, Veronica Bordoni, Evangelo Boumis, Marta Branca, Rosanna Buonomo, Donatella Busso, Marta Camici, Paolo Campioni, Flaminia Canichella, Maria Rosaria Capobianchi, Alessandro Capone, Cinzia Caporale, Emanuela Caraffa, Ilaria Caravella, Fabrizio Carletti, Concetta Castilletti, Adriana Cataldo, Stefano Cerilli, Carlotta Cerva, Roberta Chiappini, Pierangelo Chinello, Maria Assunta Cianfarani, Carmine Ciaralli, Claudia Cimaglia, Nicola Cinicola, Veronica Ciotti, Stefania Cicalini, Francesca Colavita, Angela Corpolongo, Massimo Cristofaro, Salvatore Curiale, Alessandra D’Abramo, Cristina Dantimi, Alessia De Angelis, Giada De Angelis, Maria Grazia De Palo, Federico De Zottis, Virginia Di Bari, Rachele Di Lorenzo, Federica Di Stefano, Gianpiero D’Offizi, Davide Donno, Francesca Evangelista, Francesca Faraglia, Anna Farina, Federica Ferraro, Lorena Fiorentini, Andrea Frustaci, Matteo Fusetti, Vincenzo Galati, Roberta Gagliardini, Paola Gallì, Gabriele Garotto, Ilaria Gaviano, Saba Gebremeskel Tekle, Maria Letizia Giancola, Filippo Giansante, Emanuela Giombini, Guido Granata, Maria Cristina Greci, Elisabetta Grilli, Susanna Grisetti, Gina Gualano, Fabio Iacomi, Marta Iaconi, Giuseppina Iannicelli, Carlo Inversi, Giuseppe Ippolito, Eleonora Lalle, Maria Elena Lamanna, Simone Lanini, Daniele Lapa, Luciana Lepore, Raffaella Libertone, Raffaella Lionetti, Giuseppina Liuzzi, Laura Loiacono, Andrea Lucia, Franco Lufrani, Manuela Macchione, Gaetano Maffongelli, Alessandra Marani, Luisa Marchioni, Andrea Mariano, Maria Cristina Marini, Micaela Maritti, Annelisa Mastrobattista, Ilaria Mastrorosa, Giulia Matusali, Valentina Mazzotta, Paola Mencarini, Silvia Meschi, Francesco Messina, Sibiana Micarelli, Giulia Mogavero, Annalisa Mondi, Marzia Montalbano, Chiara Montaldo, Silvia Mosti, Silvia Murachelli, Maria Musso, Michela Nardi, Assunta Navarra, Emanuele Nicastri, Martina Nocioni, Pasquale Noto, Roberto Noto, Alessandra Oliva, Ilaria Onnis, Sandrine Ottou, Claudia Palazzolo, Emanuele Pallini, Fabrizio Palmieri, Giulio Palombi, Carlo Pareo, Virgilio Passeri, Federico Pelliccioni, Giovanna Penna, Antonella Petrecchia, Ada Petrone, Nicola Petrosillo, Elisa Pianura, Carmela Pinnetti, Maria Pisciotta, Pierluca Piselli, Silvia Pittalis, Agostina Pontarelli, Costanza Proietti, Vincenzo Puro, Paolo Migliorisi Ramazzini, Alessia Rianda, Gabriele Rinonapoli, Silvia Rosati, Dorotea Rubino, Martina Rueca, Alberto Ruggeri, Alessandra Sacchi, Alessandro Sampaolesi, Francesco Sanasi, Carmen Santagata, Alessandra Scarabello, Silvana Scarcia, Vincenzo Schininà, Paola Scognamiglio, Laura Scorzolini, Giulia Stazi, Giacomo Strano,Fabrizio Taglietti, Chiara Taibi, Giorgia Taloni, Tetaj Nardi, Roberto Tonnarini, Simone Topino, Martina Tozzi, Francesco Vaia, Francesco Vairo, Maria Beatrice Valli, Alessandra Vergori, Laura Vincenzi, Ubaldo Visco-Comandini, Serena Vita, Pietro Vittozzi, Mauro Zaccarelli, Antonella Zanetti and Sara Zito.
